# Comparative Study of Markerless Vision-Based Gait Analyses for Person Re-Identification

**DOI:** 10.3390/s21248208

**Published:** 2021-12-08

**Authors:** Jaerock Kwon, Yunju Lee, Jehyung Lee

**Affiliations:** 1 Department of Electrical and Computer Engineering, University of Michigan-Dearborn, Dearborn, MI 48128, USA; 2 School of Engineering, Department of Physical Therapy and Athletic Training, Grand Valley State University, Grand Rapids, MI 49504, USA; leeyun@gvsu.edu; 3 NAVER Corp., Seongnam-si 13561, Korea; trevor0513@gmail.com

**Keywords:** markerless, vision-based, machine learning, person re-identification, gait, gait analysis, motion capture, siamese neural networks

## Abstract

The model-based gait analysis of kinematic characteristics of the human body has been used to identify individuals. To extract gait features, spatiotemporal changes of anatomical landmarks of the human body in 3D were preferable. Without special lab settings, 2D images were easily acquired by monocular video cameras in real-world settings. The 2D and 3D locations of key joint positions were estimated by the 2D and 3D pose estimators. Then, the 3D joint positions can be estimated from the 2D image sequences in human gait. Yet, it has been challenging to have the exact gait features of a person due to viewpoint variance and occlusion of body parts in the 2D images. In the study, we conducted a comparative study of two different approaches: feature-based and spatiotemporal-based viewpoint invariant person re-identification using gait patterns. The first method is to use gait features extracted from time-series 3D joint positions to identify an individual. The second method uses a neural network, a Siamese Long Short Term Memory (LSTM) network with the 3D spatiotemporal changes of key joint positions in a gait cycle to classify an individual without extracting gait features. To validate and compare these two methods, we conducted experiments with two open datasets of the MARS and CASIA-A datasets. The results show that the Siamese LSTM outperforms the gait feature-based approaches on the MARS dataset by 20% and 55% on the CASIA-A dataset. The results show that feature-based gait analysis using 2D and 3D pose estimators is premature. As a future study, we suggest developing large-scale human gait datasets and designing accurate 2D and 3D joint position estimators specifically for gait patterns. We expect that the current comparative study and the future work could contribute to rehabilitation study, forensic gait analysis and early detection of neurological disorders.

## 1. Introduction

Human gait is a noninvasive biometric feature that can be perceived from a distance, and contact with subjects is not required [1]. There have been mounting studies that show that the individuality of a subject is embedded in their gait, comprising spatiotemporal features of the ankle, knee, pelvis, and trunk [2,3,4,5]. Yet, extracting dynamic gait patterns is challenging because spatiotemporal gait representations are not easily acquired in real-world settings. In general, two main approaches were taken for gait-based identification, namely model-free (appearance-based) and model-based methods [6]. Model-free approaches are to identify an individual using one’s silhouette, clothes, anthropometrics, etc. Even with very high accuracy, using appearances has many disadvantages. Appearance-based methods must assume a subject wears unique clothes from others and works only in similar time frames when a subject does not change clothes. Using silhouettes or Gait Energy Image (GEI) [7] can mitigate the aforementioned problem. However, GEI inherently depends on shape and movement. Thus, it suffers from viewpoint variance unless a system provides all direction reference datasets. Model-based approaches consider the kinematic characteristics of gaits. To acquire such characteristics, the identification of anatomical landmarks is a prerequisite. Once the landmarks (key joint positions) are identified successfully, model-based approaches do not suffer from appearance variance. Model-based approaches can be further categorized as marker-based and markerless. Optoelectronic marker-based systems such as Vicon Nexus are much more accurate and viewpoint invariant. Yet, they require specially designed and carefully configured camera settings in a lab and markers on a subject. These limitations prevent us from using the system in real-world settings such as outdoor sports activities. Thus, there have been mounting efforts to develop markerless systems [8]. Earlier works in this lineage were not very successful due to large errors of the hip, knee, and ankle angles in their abduction/adduction/flexion/extension. As machine learning-based computer vision technologies emerge, the accuracies of markerless systems have been improved [8]. Also, much research has been done using RGB-depth camera such as Microsoft Kinect and Intel RealSense to extract kinematic information  [9,10,11,12,13,14]. Using depth data in extracting kinematic information is beneficial to improving accuracy. But RGB-depth cameras have a short ideal range (less than 5 m) for depth, so they are not common in real-world environments. According to an in depth review of markerless systems [8], most of the currently available ones are still laboratory-based or have some limitations in capture environments. Thus, in real-world settings, both marker-based and current markerless systems are not feasible options. Yang’s work on the kinematic research of swim-start on a start block can be a good example [15].

To address the aforementioned problems, we propose a novel approach where neural network-based machine learning technologies are used to infer 3D key joint positions in 2D video image sequences and extract gait cycles to re-identify a person.

**Figure 1:**
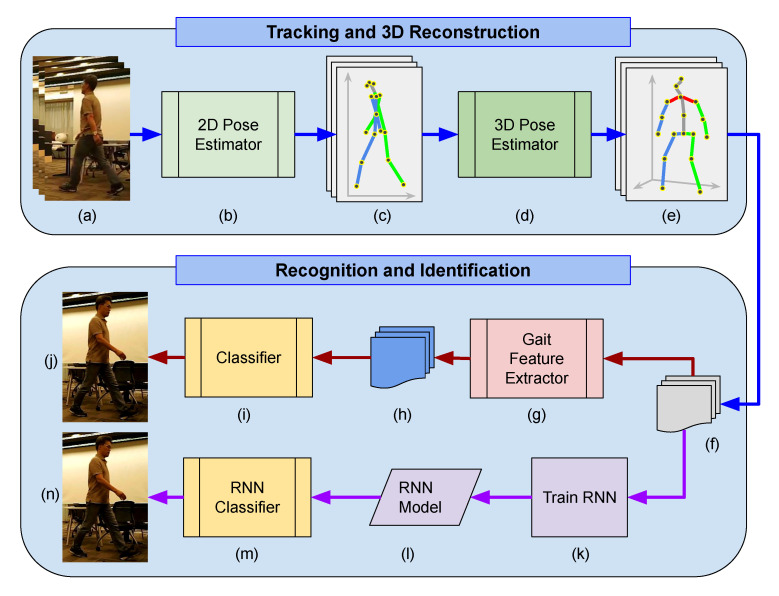
System Overview. The proposed system has two sub-sections: 3D pose estimator from 2D video input (top), gait feature extractor and classifier, and time-series-based Recurrent Neural Network (RNN) classifier (bottom). (**a**) 2D input video, (**b**) 2D pose estimator, (**c**) extracted 2D joint points with skeletal data of human pose, (**d**) 3D pose estimator, (**e**) 3D joint points, (**f**) processed 3D joint points, (**g**) gait feature extractor, (**h**) gait feature sets, (**i**) classifier based on gait features, (**j**) classified individual, (**k**) RNN trainer using spatiotemporal joint data, (**l**) trained RN model, (**m**) classifier using the RNN model, and (**n**) identified individual.

The overall system is depicted in Figure 1. There are two subsections in the system. First, 2D/3D joint estimators are to infer key joint positions in 3D from images to extract kinematic information of a subject. Second, spatiotemporal 3D key joints are fed to two different person re-identification methods: gait feature-based and spatiotemporal-based. The gait feature-based approach is to extract all possible feature values representing a person’s gait (see Section 3.5.3 for more details on gait feature sets). Then, the feature values are used to train decision tree-based classifiers that identify a person based on the gait features. The feature-free spatiotemporal method does not extract any gait features but only uses spatiotemporal changes of 3D key joints over time. The spatiotemporal data are used to train a recurrent neural network to classify a person’s gait.

The main objectives of the current study are threefold: (i) a design proposal of markerless vision-based gait analyses, (ii) validations of the proposed gait analyses in person re-identification, and (iii) a comparative study of classifiers in both feature-based gait pattern and spatiotemporal joint position-based gait patterns.

## 2. Related Work

In this section, we explore gait analyses, person re-identification, and vision-based key joint estimation.

### 2.1. Gait Analysis

To identify an individual by analyzing gait patterns, two main categories of research have been conducted: model-free and model-based approaches. One of the commonly used methods in model-free approaches is to extract human silhouettes from videos over one gait cycle and superimpose them, which is known as GEI. However, due to the body shape dependency, the approach is vulnerable to appearance changes [16]. In this category, to achieve viewpoint invariance, Das Choudhry and Tjahjadi [17] proposed to use a two-phase method based on GEI. In the first phase, candidates are filtered out using entropy. They then performed shape analysis afterward to ensure robustness to clothing variations. Zend and Wang [18] used periodic deformation of binarized silhouette models to achieve viewpoint-invariance. Yet, the gait dynamics of an individual observed in several different views must be available, which is not feasible in real-world settings.

The model-based approaches use the kinematic characteristics of the human body. Bouchrika and Nixon proposed to parameterize the motion of human joints using the elliptic Fourier descriptors [19]. The experimental results were from only the sagittal view of human walking patterns. Thus, this method will suffer from viewpoint changes. To achieve viewpoint invariance in model-based approaches, researchers used RGB-D cameras to capture 3D joint positions. Andersson and Araújo extracted anthropometry and gait features from the data acquired via Microsoft Kinect [10]. Sinha et al. used skeleton information using Kinect to identify a person and a neural network in feature selection and classification [11]. Ahmed et al. proposed a 3D gait recognition system utilizing Kinect skeleton data and reported that the fusion of joint relative distance and angle showed promising results [12]. Another 3D gait recognition method was proposed by [20] where dynamic time warping (DTW) was utilized to achieve the identification. Jiang et al. also used DTW with Kinect skeleton features but used the nearest neighbor classifier to maximize accuracy in real-time [13]. Sun et al. proposed a view-invariant gait recognition scheme where Kinect skeleton data were investigated in terms of static and dynamic features [14]. Despite recent active research in model-based gait analysis using RGB-D sensors, using such special cameras is not feasible in real-world applications since most available video clips are taken by a single RGB camera. Even if RGB-D sensors were available and captured human gait patterns, the reliable distance of depth data from such sensors is less than 5 m, which cannot be used in capturing depth data of human body joints in the distance.

Compared to previous model-free and model-based approaches, our model-based gait analysis approach can achieve viewpoint invariance. Gait features will be extracted from 3D joint positions and changes in time. Therefore, person re-identification using gait patterns can be reliably conducted with spatiotemporal changes of human body joints. Appearance changes cannot be a problem as well in our proposed method since we only use anatomical landmarks of the human body.

### 2.2. Person Re-Identification

**Figure 2:**
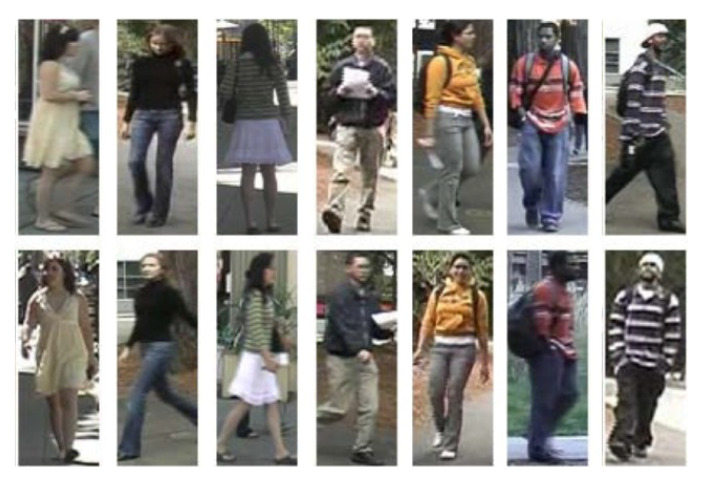
Pedestrian examples from Viewpoint Invariant Pedestrian Recognition (VIPeR) dataset. Adapted from [22]. Each column shows the same person but colors look different in other lighting conditions as in the second and last columns. The appearance-based re-identification only works with assumptions where a person shown in a camera appears in another camera in a short period of time with similar lighting conditions.

Person re-identification can be seen as multi-camera tracking. Given a person’s image, the re-identification aims to find the same person who appears over a network of cameras. Due to the changes in the lighting condition, view-angle, size of the person, and possibly different outfits, person re-identification is not a trivial problem. Figure 2 shows examples of pedestrians where each column has the same person, but colors look different in other lighting conditions. We cannot rely on GEI-based model-free approaches since the silhouettes of the same person are different, and taking the silhouettes out from the background is also challenging in real-world environments.

Appearances such as colors and styles of the outfits can be a strong cue to re-identify a person, as shown in Figure 2. However, colors can look different in different lighting conditions as in the second and last columns in Figure 2. The appearance-based re-identification only works with assumptions where a person shown in a camera appears in another camera in a short period of time with similar lighting conditions.

Several modalities such as multi-camera tracking, image-based, video-based, and end-to-end image-based have been proposed [21]. In the present study, we took a video-based re-identification with a deep learning method with a recurrent neural network. We assume that a short video clip is available where a subject walks. Our proposed work does not rely on a particular view angle, but video clips must be long enough to have multiple gait cycles. For details about gait cycles, refer to the Method section.

### 2.3. 2D and 3D Key Joints Estimation

Pose estimation in 2D from RGB input is challenging but, due to a variety of the practical applications, has been widely studied [23,24,25,26,27,28]. A common approach in 2D key joint estimation is to detect a person in an image and predict key joint positions. Convolutional Neural Networks (CNNs) are a popular choice to partition and label body parts that are then grouped together to build skeletonic figures in anthropometrically plausible manners. In the present study, our design choice for the proposed framework is Open Pose [24], which is for real-time multi-person 2D pose estimation. Part Affinity Fields (PAFs) were used to associate body parts with individuals in an image showing substantial enhancement in runtime performance and accuracy.

3D pose estimation can be categorized into single-person and multi-person estimation. Many methods in single-person pose estimation regress 3D poses by training a predictor and work well only in specific environments such as limited background and indoor setups [29]. To mitigate the problem, some took two steps instead of using direct regression of 3D poses: they estimate 2D key joint positions first and regress 3D joint positions from the 2D joints. By doing so the regression can be done more easily since the work is simplified from estimating 3D poses directly to lifting up 2D joint positions that are already found [30,31,32]. Multi-person 3D pose estimation from a single image is not as common as a single-person 3D estimation. By repeating single-person 3D estimation, a multi-person estimation can be done. Yet, holistic methods for estimating multi-person 3D poses from a single image are still needed in severe person-person occlusion cases where individual estimation suffers from the lack of information.

Recent fast technical advancement in computer vision and deep learning areas makes a certain method obsolete in less than a few years. 3D pose estimation involves several integrated tasks proposed by several research groups. The component methods needed for 3D pose estimation often make faster and more innovative advancement [33,34,35,36,37]. Holistic methods where multiple tasks are conducted simultaneously by one research group are not easy to catch up individual and specialized research groups for component methods. Thus, the present study made a critical design choice to use a step-by-step and modular approach. We chose a 3D human pose estimation method [32] and replaced its 2D key joint detection module with OpenPose [24] for a better 2D pose estimation. To validate the proposed method, we also conducted a comparative study in person re-identification using gait analyses. First, a feature-based gait analysis was performed. After extracting key joint positions from each image, gait cycles were extracted from a sequence of images. Gait features such as joint angles at a different moment in a phase of a gait cycle and relative lengths of body segments were used to discriminate an individual’s gait patterns from others. Second, without extracting such joint and body features, spatiotemporal changes of key joints were simply fed to a recurrent neural network to train to identify a person. The comparative study suggests that time-series-based classification outperforms feature-based approaches.

## 3. Method and Problem Formulation

To achieve person re-identification using markerless vision-based gait analyses, five major steps must be taken for time-series video images in sequence: (i) 2D pose estimation, (ii) 3D pose estimation, (iii) repetition of (i) and (ii) to collect several gait cycles from a subject, (iv) features extraction from gaits, and (v) person classification based on the feature-based or spatiotemporal-based method.

The proposed method is an integrated framework where key body joints are detected by a 2D pose estimator and a 3D human pose estimator restores the missing depth dimension from the 2D key body joint positions. The joint positions in 3D are inherently viewpoint invariant so that they are also free from occlusion. To conduct comparative analyses, we developed a gait feature extraction algorithm from 3D joint positions and designed a Siamese LSTM network to train a classifier for the spatiotemporal-based gait patterns. Unlike other holistic methods [31,32,38], we took a modular approach so that any individual module can be replaced with a state-of-the-art method that will be available. We chose OpenPose [24] as a 2D pose estimator since it supports 2D real-time multi-person keypoint detection. The output format of the 2D pose estimator is JSON [39]. The output JSON file is fed to a 3D pose estimator that predicts 3D joint locations using a deep end-to-end system. We chose a Simple Yet Effective Baseline (SYEB) [32] method for 3D human pose estimation. The SYEB is also a holistic method that embeds a 2D pose estimator using a stacked hourglass network [25]. We replaced the embedded 2D position detector with OpenPose, since the 2D estimator in OpenPose outperforms the embedded 2D joint position detector. After successful inference of 3D joint positions, gait analyses take place to get the re-identification system started.

### 3.1. 2D Pose Estimation from 2D Video Images

As we discussed in the Section 2.3, most 3D pose estimation methods are holistic [31,32,33,34,35,36,37]. A high quality 2D pose estimation is a prerequisite of 3D pose estimation since the estimated 2D joint locations are inputted to the 3D estimator. In this study, we took a modular design approach, where a sub-module can be easily replaced with a better method.

Formally, given a 2D RGB image *I* of size W×H at frame *t*, and the total number of images is *T*, and the input images can be described as (1).

(1) $$I={\left\{I_{t}\right\}}_{t=1}^{T},\;I_{t}\in R^{W\times H\times 3}.$$

We need a mapping function to estimate body joints in 2D from images. OpenPose is a realtime multi-person 2D pose estimation method that shows the state-of-the-art performance [24]. This method was the first place finisher in the COCO 2016 keypoints challenge. We chose to use OpenPose as a 2D pose estimator due to its performance and efficiency. The latest version of OpenPose has 3D real-time single-person keypoint detection. But this 3D keypoint detection is conducted by 3D triangulation from multiple single views, which requires multiple viewpoint views for a single person. Since the proposed work does not require multi-viewpoint images, we excluded the 3D keypoint detection of OpenPose. The 2D pose estimator of OpenPose can be saved as the JSON file format so that the 2D key joint positions can be easily transformed to an input type to a 3D pose estimator. We define estimated 2D joint positions from an image as (2).

(2) $$W={\left\{W_{t}\right\}}_{t=1}^{T},\;W_{t}\in R^{J_{2}\times 2},$$

where $J_{2}$ is the number of 2D joints.

When we define a mapping from a 2D image to 2D joint positions as: $f^{*}:R^{W\times H\times 3}\mapsto R^{J_{2}\times 2}$, Equation (3) represents a 2D joint estimator

(3) $$f^{*}=\min_{f}\frac{1}{T}\sum_{t=1}^{T}D\left(f\left(I_{t}\right),\;W_{t}\right),$$

where D(a,b) is the distance between *a* and *b*.

### 3.2. 3D Pose Estimation from 2D Pose Keypoints

3D joint positions can also be similarly defined as (4).

(4) $$S={\left\{S_{t}\right\}}_{t=1}^{T},\;S_{t}\in R^{J_{3}\times 3},$$

where $S_{t}={\left\{S_{t}\left(j\right)\right\}}_{j=1}^{J_{3}}$ and $J_{3}$ is the number of 3D joints.

When we define a mapping from 2D joint positions to 3D: $g^{*}:R^{J_{2}\times 2}\mapsto R^{J_{3}\times 3}$, Equation (5) represents a 3D estimator. The 2D joint positions from (3) are fed to a 3D joint estimator represented as in (5).

(5) $$g^{*}=\min_{g}\frac{1}{T}\sum_{t=1}^{T}D\left(g\left(f^{*}\left(I_{t}\right)\right),\;S_{t}\right).$$

We chose to use the Simple Yet Effective *Baseline* for 3D human pose estimation (SYEB) [32]. The original goal of the work was to understand error sources in 3D pose estimation from 2D images. The inference of 3D joint locations is a cascading work from 2D to 3D pose estimation. Martinez et al. wanted to identify the sources of error in the processing pipeline. What they found was that a relatively simple DNN can *lift* ground truth 2D joint locations to 3D space. Their design choice was to use 2D pose key points as input and 3D points as output. The input data format is not raw images but 2D joint locations. This is beneficial for our modular design approach since we can plug their 3D pose estimator into our process pipeline. The original 2D detection of the SYEB is the stacked hourglass network [25] pre-trained on the MPII dataset [23]. We replaced this 2D pose detection with OpenPose 2D pose estimation since OpenPose shows higher accuracy in 2D pose estimation.

The human joint parts are differently indexed in OpenPose and the stacked hourglass network. OpenPose uses COCO dataset parts index numbers (eighteen 2D keypoints) and the stacked hourglass network uses the H3.6M dataset [40] parts index numbers (thirty-two 3D keypoints).

- The joint index numbers of the COCO dataset are as follows: 0-nose, 1-neck, 2-right shoulder, 3-right elbow, 4-right wrist, 5-left shoulder, 6-left elbow, 7-left wrist, 8-right hip, 9-right knee, 10-right ankle, 11-left hip, 12-left knee, 13-left ankle, 14-right eye, 15-left eye, 16-right ear, 17-left ear.
- The join index numbers of H3.6M dataset are as follows: 0-midpoint of the hips, 1-right hip, 2-right knee, 3-right ankle (foot), 6-left hip, 7-left knee, 8-left ankle, 12-spine, 13-thorax, 14-neck/nose, 15-head, 17-left shoulder, 18-left elbow, 19-left wrist, 25-right shoulder, 26-right elbow, 27-right wrist.

**Figure 3:**
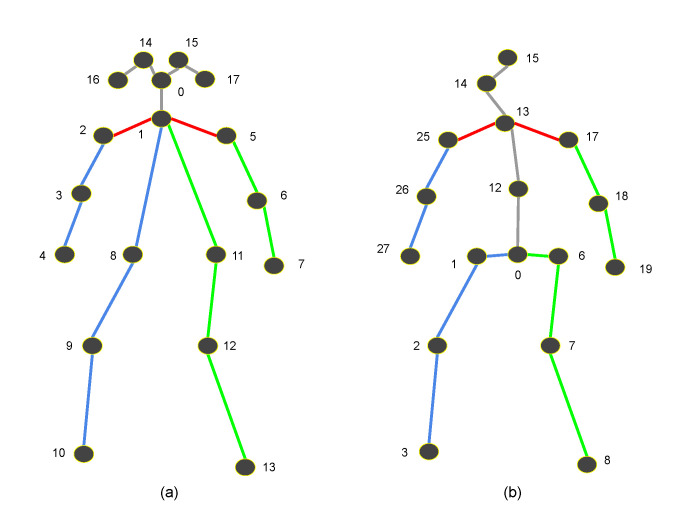
Skeletal model difference. (**a**) COCO dataset parts index numbers. (**b**) H3.6M dataset parts index numbers.

The differences indicate that the output of OpenPose must be converted to a proper format for the subsequent 3D pose estimation that uses the H3.6M dataset parts index numbers. In addition to the index number reassignment, additional locations such as the head, nose, neck, spine, and the center of the hip must be calculated using neighboring positions and added to the list of joint positions. A 2D point for the center of the hip must be generated with the left and right hip positions. The index number 12 in the H3.6M dataset parts does not exist in the COCO dataset index. The approximation has to be made using the hip center and neck. We used the center position between them as a position of index 12 (spine in H3.6M dataset parts index). See more details about the differences in Figure 3.

**Figure 4:**
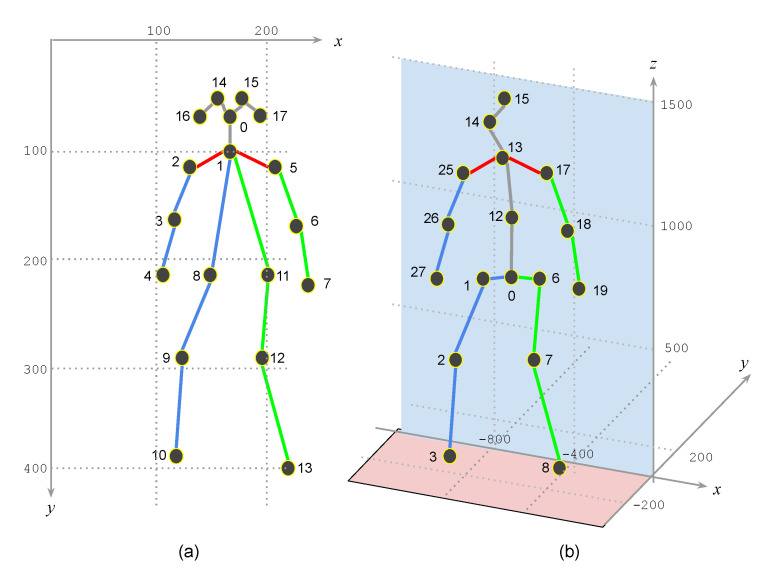
The coordinate system difference between (**a**) OpenPose. The joint positions are labeled in 2D locations. (**b**) SYEB. The joint positions are in 3D. The depth information is inferred by the embedded 3D joint position estimator.

The coordinate system also has differences. The 2D positions from OpenPose have their coordinate origin at the left top corner. The 3D positions from the SYEB have their coordinate origin at the right bottom side. The z-axis is from the bottom to the top. The *y*-position of the joint 0 is 0. The 3D joint positions are not determined by their corresponding 2D positions. The detected positions do not have a unit. See the coordinate system difference in Figure 4.

### 3.3. Gait Features

**Figure 5:**
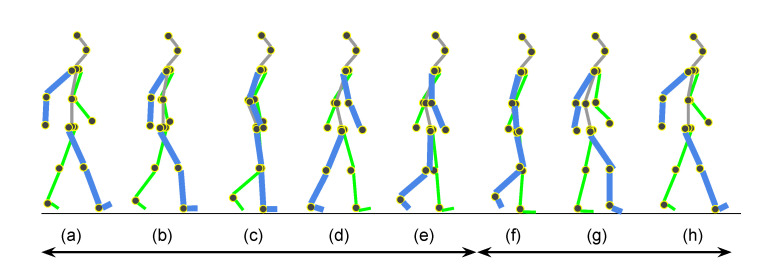
Human gait cycle. (**a**) Initial contact, (**b**) Loading response, (**c**) Mid-stand, (**d**) Terminal stance, (**e**) Pre-swing, (**f**) Initial swing, (**g**) Mid-swing, (**h**) Terminal swing. The stance phase is defined from (**a**) to (**e**). The swing phase is from (**f**) to (**h**). The initial contact and the terminal swing is the same event with a different name.

After successful 3D pose estimation, gait patterns will be analyzed to re-identify a person. Gait is defined as locomotion that can be achieved through the movement of limbs. Human gait is bipedal using forward propulsion of the center of gravity (CoG) of the body. A certain pattern is repeated during the movement. We define the repeated pattern as a gait cycle. A gait cycle refers to the sequence of events during locomotion. A bipedal gait has two phases: the stance phase and the swing phase. Individuals have different gait patterns since there are characteristic features in the movements of their limbs, the velocity of joints, kinetic energy, and anthropometric measures. Figure 5 shows the human gait cycle.

To extract gait patterns, a sequence of 3D joint positions is required. With $S^{\left(c\right)}={\left\{S^{\left(c\right)}\right\}}_{c=1}^{N_{c}}$ where $N_{c}$ is the number of walkers, we define the *i*-th gait sample as $G_{i}^{\left(c\right)}$ extracted from $S^{\left(c\right)}$ for the person *c* who can have multiple gait cycles. We define a group of gait cycles of the person *c* as (6).

(6) $$G^{\left(c\right)}={\left\{G_{i}^{\left(c\right)}\right\}}_{i=1}^{N_{g}^{\left(c\right)}},$$

where $N_{g}^{\left(c\right)}$ is the number of gait cycles for the person *c*.

To have $G^{\left(c\right)}$, we need a gait cycle extractor *h* shown in (7) that can be defined as a mapping from a sequence of the 3D joint positions of a subject body to gait pattern data.

(7) $$h:{\left\{S_{i}^{\left(c\right)}\right\}}_{i=1}^{N_{g}^{\left(c\right)}}\mapsto G^{\left(c\right)}.$$

Feature-based training can be described as follows. The scatter matrices for intra- and inter-class are shown in (8) and (10).

(8) $$\Sigma_{inter}=\sum_{c=1}^{N_{c}}\left(\mu^{\left(c\right)}-\mu \right){\left(\mu^{\left(c\right)}-\mu \right)}^{⊤},$$

where $\mu^{\left(c\right)}=\frac{1}{N_{g}^{\left(c\right)}}\sum_{i=1}^{Ng^{\left(c\right)}}G_{i}^{\left(c\right)}$ and $\mu =\frac{1}{N_{c}}\sum_{i=1}^{N_{c}}\mu^{\left(i\right)}$.

(9) $$\Sigma_{c}=\frac{1}{N_{c}}\sum_{i=1}^{N_{g}^{\left(c\right)}}\left(G_{i}^{\left(c\right)}-\mu^{\left(c\right)}\right){\left(G_{i}^{\left(c\right)}-\mu^{\left(c\right)}\right)}^{⊤},$$

(10) $$\Sigma_{intra}=\sum_{c=1}^{N_{c}}\Sigma_{c}.$$

The class separability was formularized as (11) in [41]. The feature-based training can be understood as maximizing the class separability of given feature space.

(11) $$\Psi =tr(\Sigma_{inter}-\Sigma_{intra}).$$

The trace function tr(m) measures the overall variance of a given matrix *m*, i.e., the larger value of tr(m) indicates more scattered in a feature space. Therefore, the training is a process of minimizing $\Sigma_{intra}$ while maximizing $\Sigma_{inter}$.

### 3.4. Gait Cycle Extraction

A gait cycle can be extracted using a simple anthropometric measure that is the Euclidean distance between the left ankle position and the right. The maximum distance can be either the initial contact (a) or the terminal swing (h) in Figure 5. The Euclidean distance of the left and right ankle is defined as follows.

(12) $$d_{t}\left(a\right)=\left|S_{t}\left(3\right)-S_{t}\left(8\right)\right|.$$

**Figure 6:**
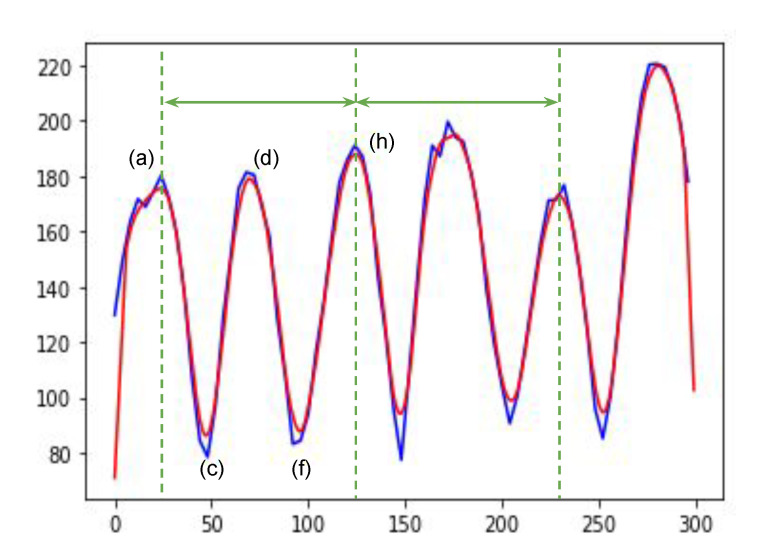
Gait cycle extraction in a sample of $d_{t}\left(a\right)$. One gait cycle (green solid line arrows) is from the changes in the distance of two ankles. To remove a subtle change in these ankle distances, data smoothing is applied. The line in blue is raw data. The line in red is after smoothing. The *x*-axis is for the image frame numbers. The *y*-axis is for the 3D distances between the two joints.

The part index number 3 is the right ankle index number, and 8 is the left. The local maxima of the $d_{t}\left(a\right)$ are considered as the moments where two ankles have the maximum distance. In Figure 5, the initial contact (a) (=terminal swing (h)) and terminal stance are the moments when two ankles have the maximum distance. Therefore, a gait cycle can be identified with a sequence of one local maximum, one local minimum, and another local maximum. Figure 6 shows an example of the Euclidean distances between two ankles. Based on the local maxima, we can extract gait cycles from time-series data points of 3D joint positions. This problem looks trivial, but it needs extra data preprocessing steps due to the pose estimation errors and the occasions where there are not enough data acquisition frequencies. As we can see in Figure 6, there are multiple local maxima around the peaks (see the solid blue line). In the next section, we will explain the data preprocessing in more detail.

#### 3.4.1. Interpolation and Smoothing

Due to the small frame numbers per second, the image sequences may not have critical moments in gait cycles. Thus, it was imperative for us to add additional 3D key joint points between frames. We interpolated the raw 3D joint points with four more points using the Cubic Spline interpolation.

**Figure 7:**
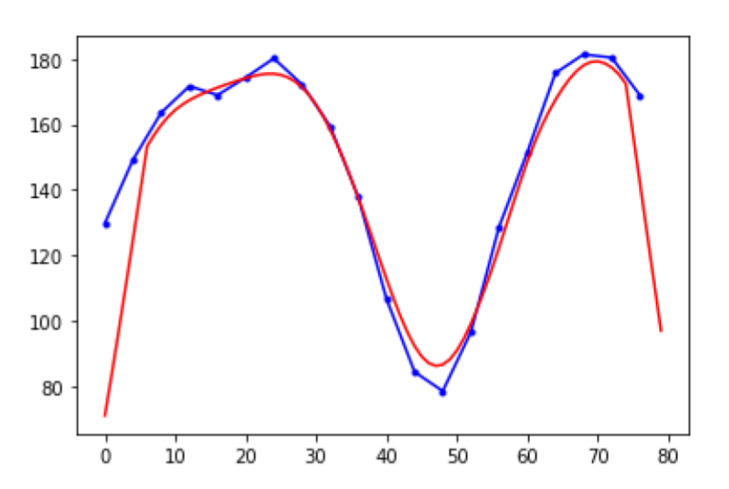
An example of data smoothing using discrete linear convolution.

A smoothing process must be subsequently applied to the interpolated raw key joint points to make clear local maxima and minima. To implement a smoothing process, we used a discrete linear convolution.

(13) $$\left(d*b\right)\left[n\right]=\sum_{m=0}^{M}d\left[n-m\right]b\left[m\right],$$

where *d* is an array of Euclidean distances, *b* is a moving average filter, and *M* is the filer size. We used 12 for *M*. Figure 7 shows an example of data smoothing using discrete linear convolution.

#### 3.4.2. Getting Local Maxima and Minima

Maxima and minima can be identified at points where the derivatives are zeros. Also, we can distinguish maxima and minima from the identified points by getting their second derivatives. But due to the slight data fluctuations, this basic method is not sufficiently reliable. Therefore, we used the following algorithms to get local maxima and minima from the distances of both ankles. We used 7 for *N* (window size).

- Create a moving window. Let *N* be the window size.
- From the center point, calculate the slopes of all left N/2 points.
- From the center point, calculate the slopes of all right N/2 points.
- If all left slopes are positive and all right slopes are negative, then the center point is a local maximum.
- If all left slopes are negative and all right slopes are positive, then the center point is a local minimum.

### 3.5. Feature-Based Approach

A feature-based approach can be categorized into two: dynamic and anthropometric static features.

#### 3.5.1. Dynamic Gait Features

**Figure 8:**
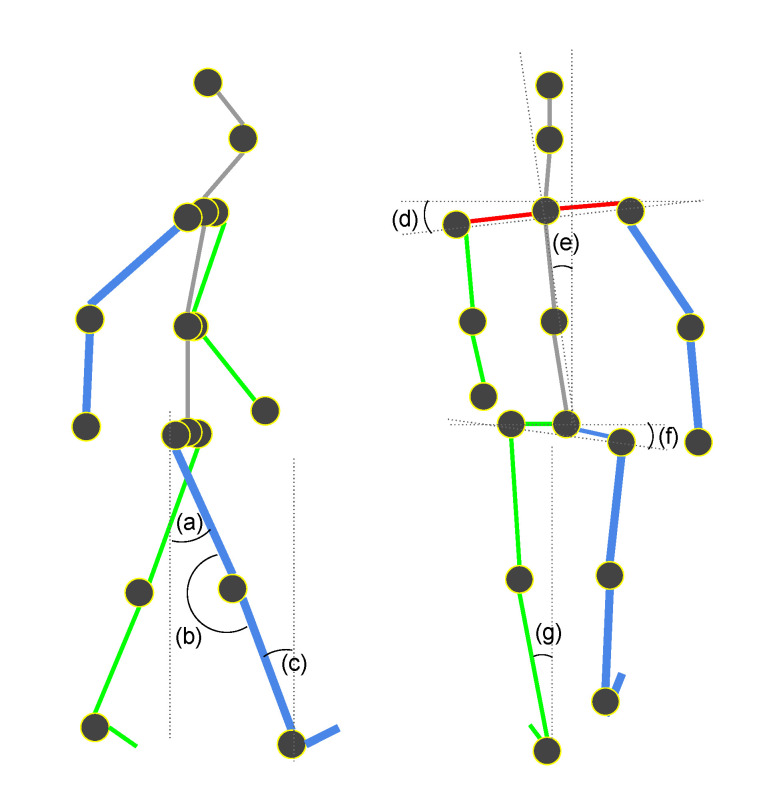
Angle features. (**a**) Hip extension angle, (**b**) Knee flexion angle, (**c**) Leg inclination angle, (**d**) Lateral shoulder drop angle, (**e**) Trunk side bending angle, (**f**) Lateral pelvic drop angle, (**g**) Rearfoot eversion angle.

We used mainly the lower body for the features since the positions of the upper limbs are not consistent during walking [42]. Features can be categorized with angles, lengths, areas, and times at a certain moment during a gait cycle. Angle features can be defined as described in Figure 8. These angle features can be used at a certain moment during a gait cycle. This will guarantee that we compare the same angle features at the same moment. At the initial contact during a gait cycle, hip extension, knee flexion, leg inclination, trunk side bending, lateral shoulder drop, lateral pelvic drop, and rearfoot eversion are potential gait features. At the mid-stand, knee flexion is a gait feature. At the pre-swing, hip extension angle and leg inclination angle are gait features.

In a gait cycle, we can also mean, standard deviation, maximum and minimum values of certain angles. The following three angles can be considered to extract features from a gait cycle [42].

- The angles of the upper leg (thigh) relative to the vertical.
- The angles of the lower leg (calf) relative to the upper leg (thigh).
- The angles of the ankle relative to the horizontal.

Also, the following values can be useful features in a gait cycle.

- The means and standard deviations of the horizontal and vertical distances between the feet and knees and between the knees and shoulders.
- The mean areas of the triangle of the root ($S_{t}\left(0\right)$) and two feet.
- The step length: the maximum distance between two feet.

The following time-related features can be also a plus.

- The gait cycle time: from (a) to (h) in Figure 5.
- The gait velocity: two times of a step length is divided by a gait cycle.

#### 3.5.2. Anthropometric Static Features

Anthropometric features are not changing during a gait cycle. Thus, they can be considered static features. Yet, due to the range of inference errors during 2D pose estimation and subsequent 3D pose estimation, it would be ideal to use the mean values of these lengths during a gait cycle. Anthropometric features are the lengths of two neighboring joints. These lengths must be adjusted with a reference length since the size of a person in an image can vary. In this research, no reference length was provided in the dataset that we used. Thus, we were not able to use anthropometric features as they are. Instead, we used a ratio of the lengths between two relevant joints.

#### 3.5.3. Gait Feature Sets

This section describes the gait features that we selected for this comparative study. The features that we used are angle, length, and time.

Joint angle features can be used to identify an individual since they show different characteristics among individuals. We first need to redefine the time frame from the simple image index to the gait phase in a cycle. $t=\{t_{a},t_{b},\ldots ,t_{h}|a=initial\_contact,b=loading\_response,\ldots ,h=terminal\_swing\}$. Also, note that, in this study, the hip extension angle is approximated with an angle between one thigh and another thigh. The hip extension angle was originally defined as an angle between one thigh and the vertical line from the pelvis to the ground. See Figure 8a for the definition of the hip extension angle. We define $\bar{S_{t}}$ as (14) for lifting a position on the x−y plane up toward the *z* axis.

(14) $$\bar{S_{t}}=S_{t}+\left[0,0,H\right],$$

where *H* is a constant number to raise the *z*-axis assuming the ground is the x−y plane.

All angle features that we used in this study are as follows.

- **max_Rdegree**: the maximum right knee flexion
  $$=\max_{t_{a}\leq t\leq t_{h}}\left(\left\{∡\left(S_{t}\left(3\right),S_{t}\left(2\right),S_{t}\left(1\right)\right)\right\}\right)$$
- **max_Ldegree**: the maximum left knee flexion)
  $$=\max_{t_{a}\leq t\leq t_{h}}\left(\left\{∡\left(S_{t}\left(7\right),S_{t}\left(6\right),S_{t}\left(8\right)\right)\right\}\right)$$
- **min_Rdegree**: the minimum right knee flexion
  $$=\min_{t_{a}\leq t\leq t_{h}}\left(\left\{∡\left(S_{t}\left(3\right),S_{t}\left(2\right),S_{t}\left(1\right)\right)\right\}\right)$$
- **min_Ldegree**: the minimum left knee flexion
  $$=\min_{t_{a}\leq t\leq t_{h}}\left(\left\{∡\left(S_{t}\left(7\right),S_{t}\left(6\right),S_{t}\left(8\right)\right)\right\}\right)$$
- **initial_contact_hip_extension**
$$=∡(S_{t_{a}}\left(7\right),S_{t_{a}}\left(6\right),S_{t_{a}}\left(3\right))$$
- **initial_contact_left_knee_extension**
$$=∡(S_{t_{a}}\left(6\right),S_{t_{a}}\left(7\right),S_{t_{a}}\left(8\right))$$
- **initial_contact_left_leg_inclination**
$$=∡(S_{t_{a}}\left(7\right),S_{t_{a}}\left(8\right),\bar{S_{t}\left(8\right)})$$
- **initial_swing_knee_flextion**
$$=∡(S_{t_{f}}\left(6\right),S_{t_{f}}\left(7\right),S_{t_{f}}\left(8\right))$$
- **mid_stance_knee_flextion**
$$∡(S_{t_{c}}\left(6\right),S_{t_{c}}\left(7\right),S_{t_{c}}\left(8\right))$$
- **terminal_stance_hip_extension**
$$=∡(S_{t_{d}}\left(7\right),S_{t_{d}}\left(6\right),S_{t_{d}}\left(2\right))$$
- **terminal_stance_right_knee_flextion**
$$=∡(S_{t_{d}}\left(1\right),S_{t_{d}}\left(2\right),S_{t_{d}}\left(3\right))$$
- **terminal_stance_right_leg_inclination**
$$=∡(S_{t_{d}}\left(2\right),S_{t_{d}}\left(3\right),\bar{S_{t_{d}}\left(3\right))}$$
- **terminal_stance_left_leg_inclination**
$$=∡(S_{t_{d}}\left(7\right),S_{t_{d}}\left(8\right),\bar{S_{t_{d}}\left(8\right)})$$
- **terminal_swing_hip_extension**
$$=∡(S_{t_{h}}\left(2\right),S_{t_{h}}\left(1\right),S_{t_{h}}\left(7\right))$$
- **terminal_swing_right_leg_inclination**
$$=∡(S_{t_{h}}\left(2\right),S_{t_{h}}\left(3\right),S_{t_{h}}\left(3\right))$$

Absolute lengths of body parts are not given in the datasets. Thus, we used relative length features instead. With the definition of D(p1,p2) that is the Euclidean distance of two joints p1 and p2, the following are the length features of a subject body.

- **upper_body**:
  $$ub=D\left(S_{t}\left(0\right),S_{t}\left(12\right)\right)+D\left(S_{t}\left(12\right),S_{t}\left(13\right)\right)$$
- **right_lower_leg**: the right calf w.r.t bb
  $$=D(S_{t}\left(3\right),S_{t}\left(2\right))/ub$$
- **right_upper_leg**: the right thigh w.r.t bb
  $$=D(S_{t}\left(2\right),S_{t}\left(1\right))/ub$$
- **left_lower_leg**: the left calf w.r.t bb
  $$=D(S_{t}\left(8\right),S_{t}\left(7\right))/ub$$
- **left_upper_leg**: the left thigh w.r.t bb
  $$=D(S_{t}\left(7\right),S_{t}\left(6\right))/ub$$

We also used dynamic features that can be defined as follows. The left and right stride are dynamic features that must be calculated from the gait cycle shown in Figure 5.

- **left_stride** = $D(S_{t_{a}}\left(8\right),S_{t_{d}}\left(8\right))$
- **right_stride** = $D(S_{t_{d}}\left(3\right),S_{t_{h}}\left(3\right))$

Time-related features are defined as follows.

- **RFoot_period**: frames where the right foot is ahead of the left.
- **LFoot_period**: frames where the left foot is ahead of the right.
- **period**: the total frames in a gait cycle.

### 3.6. Spatiotemporal-Based Approach

Another way to extract representations from a gait pattern is to use changes of 3D joint positions in time. We used the following seven 3D locations of key joints in a gait.

- Left ankle, knee, and hip: $S_{t}\left(8\right)$, $S_{t}\left(7\right)$, $S_{t}\left(6\right)$
- Right ankle, knee, and hip: $S_{t}\left(3\right)$, $S_{t}\left(2\right)$, $S_{t}\left(1\right)$

### 3.7. Classification

In this study, we used several different classification methods that fall into two categories. The first category of the methods is the Ensemble method that uses multiple predictors inside to improve accuracy. The second is a connectionist approach to capture spatiotemporal features of gaits. We chose a recurrent neural network for this purpose.

#### 3.7.1. Feature-Based Approach

We chose three Ensemble methods: Random Forest [43], XGBoost [44] and CatBoost [45], since they allow us to see which features were more important than others. Using these classification methods is beneficial because they not only infer a class but also show the order of importance of those features. Random Forest uses a number of decision trees to improve prediction accuracy while controlling the overfitting problem. XGBoost and CatBoost are gradient boosting on decision trees. They are also able to show the feature importance in order.

#### 3.7.2. Spatiotemporal-Based Approach

**Figure 9:**
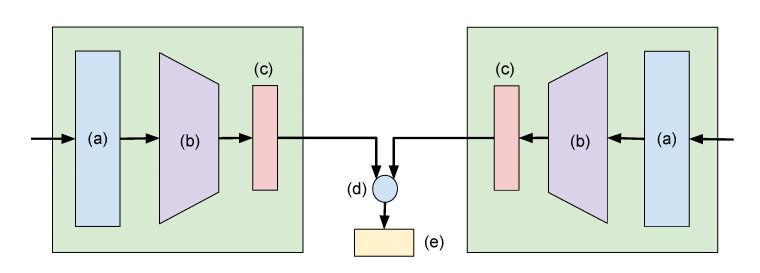
Siamese Neural Network with two identical subnetworks. (**a**) Input (**b**) Convolutional Neural Network layers (**c**) Fully connected—sigmoid layer (**d**) Absolute difference (**e**) Fully connected—sigmoid layer.

We used a Siamese LSTM network (Figure 9) with seven spatiotemporal values in this approach. A Deep Neural Network (DNN)-based learning needs ample datasets to train a huge number of network weights. In this study, we only have a few gait cycles from the MARS and CASIA-A datasets. This prohibited us from using a general DNN. Inspired by the human ability to acquire and recognize a new pattern, researchers developed a way to train a neural network with little data [46]. The Siamese network has, as its name implies, a twin network that shares the same parameter. Their outputs will be computed in the L1 component-wise differences and compared to have a similar output from the same class and a different output from the different classes.

The input to the network is a fixed size vector, $\Lambda_{t}$ as in (15) to encode the underlying features in a gait cycle.

(15) $$\Lambda_{t}=\left[S_{t}\left(8\right),S_{t}\left(7\right),S_{t}\left(6\right),S_{t}\left(3\right),S_{t}\left(2\right),S_{t}\left(1\right)\right]$$

Therefore, a gait can be defined as (16) in a spatiotemporal-based approach.

(16) $$G={\left\{\Lambda_{t}\right\}}_{t=1}^{N}$$

**Figure 10:**
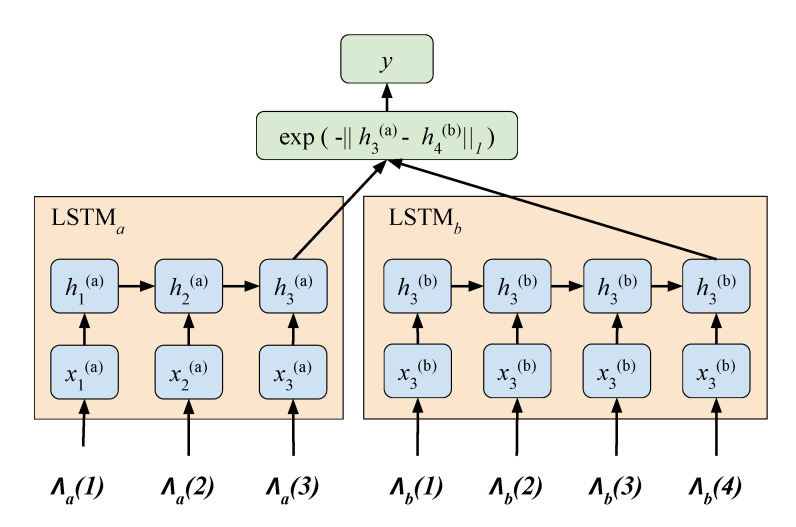
Siamese recurrent architectures for learning sentence similarity. Adapted from [47].

Our model is applied to assess gait similarity between the lower limb movement identified by the 3D key joints. We used the Manhattan LSTM (MaLSTM) model [47] shown in Figure 10.

### 3.8. Datasets

To study person re-identification using gait features, we used two datasets: MARS (Motion Analysis and Re-identification Set) [48] and CASIA (The Institute of Automation, Chinese Academy of Sciences) Dataset A [4].

**Figure 11:**
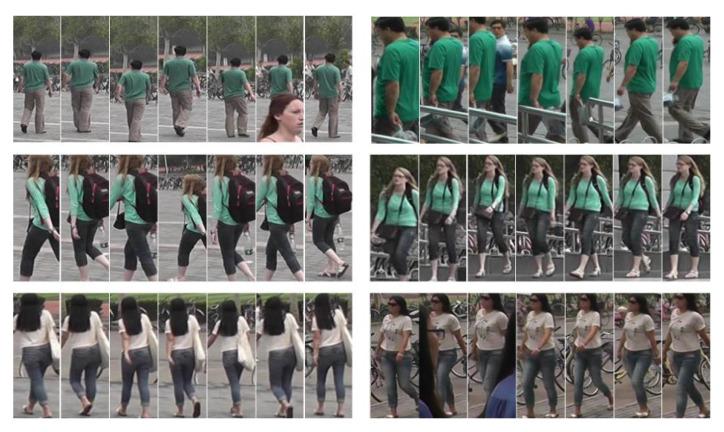
A sample tracklet of MARS. Adapted from [48]. Each row is labeled as the same identity.

The MARS was released in 2016 to be used for re-identification. This, presumably the largest video datasets for person re-identification, has around 20,000 tracklets from 1261 identities with random angles. Not all tracklets have gait data. For the current study, we removed some tracklets that do not have gaits and collected 264 subjects where we were able to extract 8 to 25 gait cycles from each class. In the present experiments, we used eight gait cycles per class. Some sample tracklets are shown in Figure 11.

**Figure 12:**
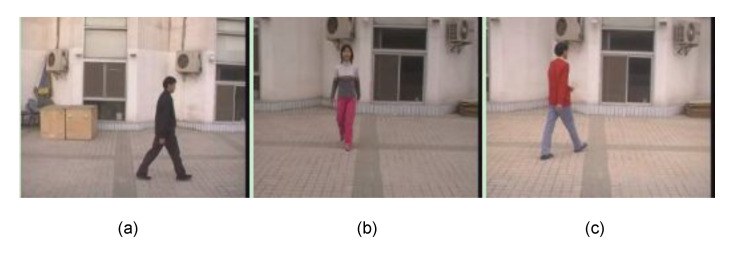
An example of CASIA Dataset A [4]. The images show three different angles of each person’s data. (**a**) Parallel to the image plane, (**b**) 90 degrees, (**c**) 45 degrees.

**Figure 13:**
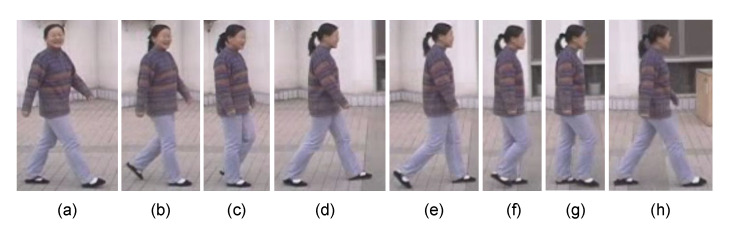
Gait example from CASIA-A dataset. (**a**) Initial contact, (**b**) Loading response, (**c**) Mid-stand, (**d**) Terminal stance, (**e**) Pre-swing, (**f**) Initial swing, (**g**) Mid-swing, (**h**) Terminal swing. The stance phase is defined from (**a**) to (**e**). The swing phase is from (**f**) to (**h**).

The CASIA Dataset A was created in 2001 and released through [4]. Some sample images are shown at Figure 12. The Dataset A includes twenty people. Three different angles (parallel, $45^{\circ }$, and $90^{\circ }$ to the image plane) were used to acquire two sets of walking image sequences. Each set has image sequences in which one person moves straight from one end position to another. This means that each person’s data comprises twelve sets of image sequences. A set of image sequences equals about three seconds of video with a rate of 25 frames/s. Figure 13 presents a gait example of the dataset.

## 4. Experimental Results

Experiments on the MARS and CASIA-A datasets show that the Siamese LSTM outperforms feature-based approaches by 20% on MARS and 55% on CASIA-A. We interpret the result as follows. The Siamese LSTM approach shows better performance because extracted gait features are not accurate enough to clearly show the individual variations in gait patterns. The 2D and 3D pose estimators we used in this study were trained with various human motion, not particularly human gaits. We assume that the pose estimators need to be enhanced to compete with the spatiotemporal-based classification powered by the Siamese LSTM network. Our future study is to enhance the accuracy of estimations of 2D and 3D joint positions in gait patterns by developing large-scale human gait datasets and designing 2D and 3D joint estimators specifically for gait patterns. The technical details of the experiments are as follows.

Python 3.7 with scikit-learn 0.22.1, xgboost 0.9, catboost 0.21, and R programming language were used for the Ensemble methods. To implement and test a Siamese LSTM, we used Keras with Tensorflow. Data preprocessing was done with R programming language. GeForce GTX 2080Ti and Intel Core i7-9800X @ 3.80 GHz×16 were used for GPU and CPU respectively. The computer operating system was 64-bit Ubuntu 18.04 LTS.

We collected 264 subjects from MARS where we were able to extract the minimum eight gait cycles from each class. From CASIA-A, we collected two different angles out of three. Due to the low-resolution of the data, the image sequences of a subject who walks from a distance to the front, named as $90^{\circ }$ in the dataset, were not able to be used. See Section 5 for more details. Each person has two round trips in each view angle. Thus, eight samples were collected per person. The number of subjects in CASIA-A is 20. This means the total number of samples is 160. We were able to extract three gait cycles from single direction walking. This allowed us to use twelve gait cycles per person.

**Figure 14:**
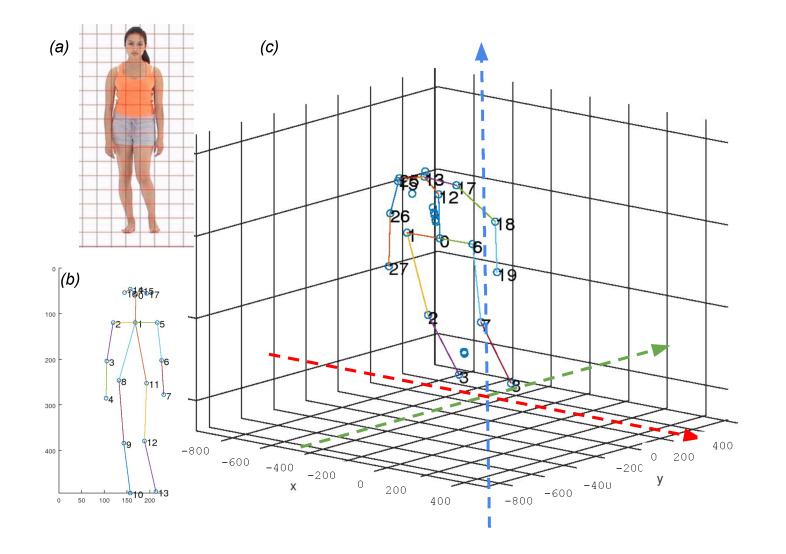
An example of 2D and 3D key joint estimation. (**a**) Input image. (**b**) The 2D estimation result from the input image. (**c**) The 3D estimation from the 2D key joints.

**Figure 15:**
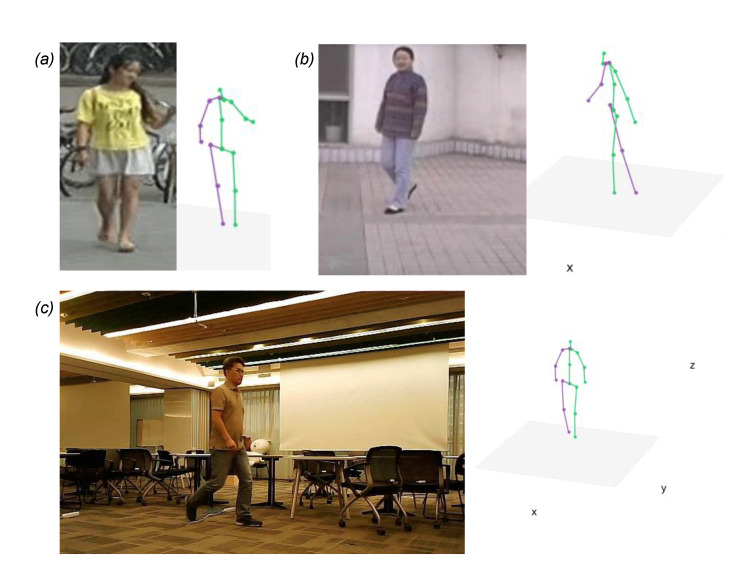
Examples of the 3D estimation. (**a**) MARS datasets. (**b**) CASIA-A datasets. (**c**) A sample from the authors.

We used 20 frames per second for MARS and 25 frames per second for CASIA-A to create videos of walking at normal speed. First, the system cranks the processing pipeline up. It reads a video and feeds it to the 2D estimator, OpenPose [24] that generates 2D joint positions in the JSON format. Then, the 3D estimator, SYEB [32] lifts up the 2D joint positions to 3D by inferring the depth information. Figure 14 shows an example of 3D key joint estimation in step by step. The 3D joint estimation in Figure 15 represents the SYEB outputs from multiple datasets. After the 3D estimator successfully predicts the joint positions in 3D, a gait cycle extractor starts identifying the gaits in the sequential data of 3D joints. Then, gait cycles are analyzed to extract gait features.

### 4.1. Training Ensemble Methods

We used three ensemble methods: Random Forest, XGBoost, and CatBoost. To use the Random Forest, we chose the R programming language that is popular for data analysis. The training and test ratio was seven to three. Two parameters must be tuned in the R: mtry and ntree. The number of variables randomly selected as candidates at each split is defined as mtry. The number of trees to grow is defined as ntree. The optimal numbers of mtry and ntree can be tuned by the tune function. We used 6 for mtry and 750 for ntree. To use XGBoost and CatBoost, we chose Python with scikit-learn [49]. The 25% data samples were randomly selected for test data. For XGBoost, we chose *gbtree* as a booster. The maximum tree depth was 3 and the number of boosting rounds was 300. For CatBoost, we used MultiClass as loss_function and 200 was chosen for iterations. We repeated each classifier 100 times and reported the mean values of accuracy and F-1 score in Table 1.

### 4.2. Training Siamese-LSTM Network

**Figure 16:**
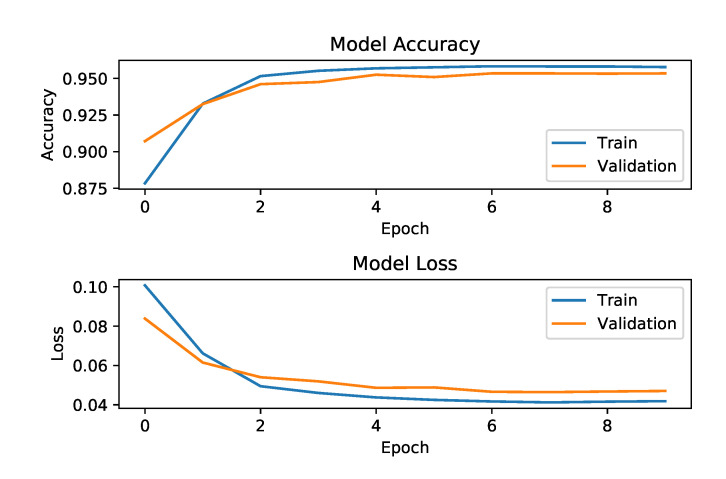
The training graphs for the Siamese-LSTM network with CASIA-A. The graph shows the training was successful with around 95% accuracy and 0.04 loss after 10 epochs.

We prepared input data for the Siamese LSTM network as follows. The estimated 3D key joint positions with temporal information from the hip, knee, and foot from both legs were used as inputs. Our LSTM uses 18 (=six joint positions in 3D space) dimensional representations $h_{t}$ and memory cells $c_{t}$. The batch size was 1024. The number of epochs was 10. The Mean Square Error (MSE) was used as a loss function. We used the Adam optimizer [50] as a parameter optimization method. The model accuracy and loss graphs are depicted in Figure 16 and strongly indicate that the training has been done successfully.

### 4.3. Classification Performance Comparison

**Figure 17:**
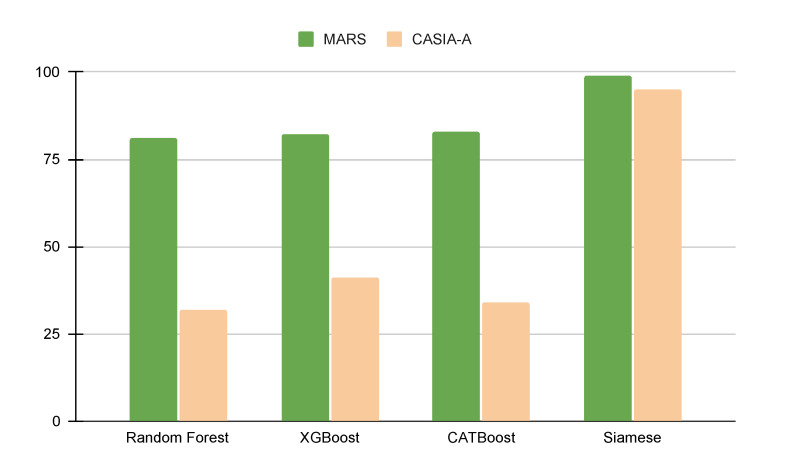
The performance of classifiers accuracy for the MARS and CASIA-A datasets.

The classification performances of gait feature-based and lower-body spatiotemporal information-based were reported in Figure 17 as well as Table 1. Siamese LSTM method using lower body spatiotemporal information outperforms the ensemble methods (Random Forest, XGBoost, and CatBoost) in two different datasets. No meaningful performance difference was observed between ensemble methods. The gait-feature-based approaches show much lower performance in CASIA-A datasets compared to MARS. This is partly because of the small size of human subjects in the dataset. The size of a subject is as small as 27×76, which makes it a challenging task for the 2D estimator to predict exact 2D joint positions. Due to the inaccurate 2D joint positions, it deteriorates the 3D estimator’s lifting to 3D performance. As a result, gait features that are sensitive to 3D joint positions were not accurate enough to classify individuals based on their gait features.

## 5. Discussion

We would like to discuss two topics: (i) separability and importance of features and (ii) open datasets for gait patterns.

### 5.1. Feature Study

**Figure 18:**
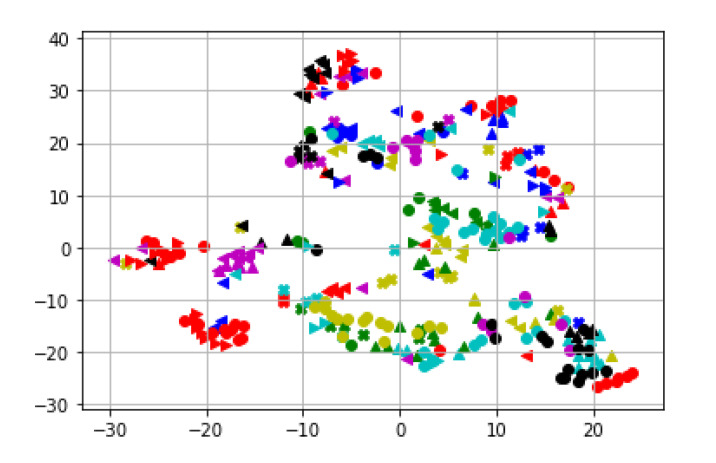
t-SNE feature maps of MARS. A different color and shape marker indicates a feature.

**Figure 19:**
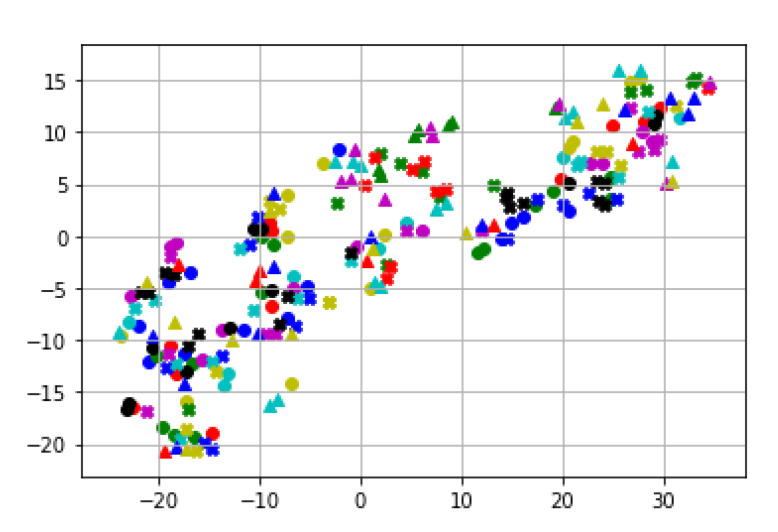
t-SNE feature maps of CASIA-A. A different color and shape marker indicates a feature.

To investigate the feature separability, we first visualized the feature maps using the t-Distributed Stochastic Neighbor Embedding (t-SNE) [51]. We used learning_rate = 200, n_iter = 700, verbose = 2, perplexity = 20 for the t-SNE visualization. Figure 18 shows the separability of the 24 gait features for MARS datasets. Note that we do not separate a feature among others, so this map does not directly represent our classification performance. Our classifier uses all the features to classify an individual. Thus, the separability in Figure 18 and Figure 19 are promising since we do not use a single feature to identify a class.

Even though the ensemble approaches showed lower performance than the Siamese LSTM with spatiotemporal information of gaits, the decision trees inside the ensemble methods can identify which features are more important than others. Table 2 reports the top ten gait features considered important in their classification tasks.

The lengths of the strides of each foot (**right_stride**, **left_stride**), speed of walking (**period**), and relative lengths of lower body parts (**left/right_upper/lower_leg**) are generally more important than angular values in a certain phase in a gait cycle (**max/min_Ldegree**, **terminal_swing/stance_extension**) according to Table 2. The results can be a strong indication that angular gait features were not accurate enough to differentiate a person from others based on the features.

### 5.2. Further Thoughts on Feature-Based Approach

**Figure 20:**
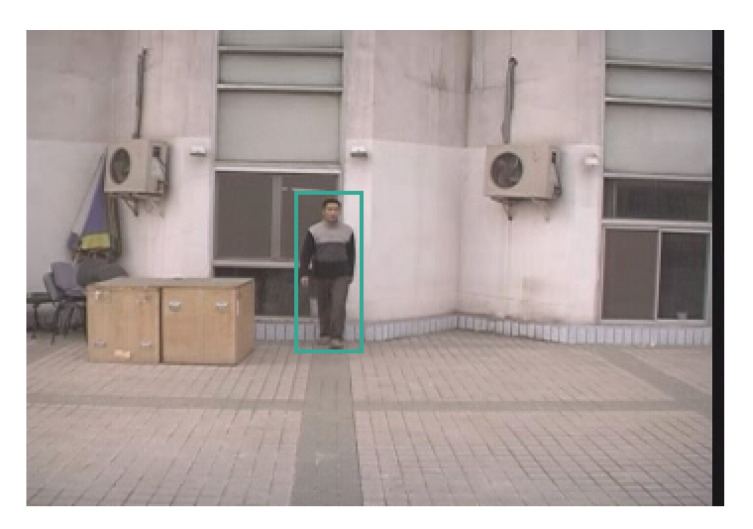
A 90° example from CASIA-A. The data name is lsl-90_1-006.png. The image size is 352×240 and the human subject size is 30×78.

From this comparative study, we found that a recurrent neural network trained by spatiotemporal lower body joint data outperformed gait feature-based ensemble methods. Yet, we believe that it is premature to conclude that a gait feature-based person re-identification approach is not a good choice. The results from the present work have the following potential limitations. The currently available human pose datasets are not specifically for human gait study. The 2D and 3D pose estimators were trained with various human poses. To increase the overall accuracy of the estimations, the prediction accuracies of some sets of human motions or poses were possibly sacrificed. Another challenge was the low-resolution of human subjects in the datasets that adversarially affect the accuracy of 2D key joint estimations. Even though the 2D pose estimator we used was successful in extracting body parts from a small (30×78) and blurred subject in an image (352×240), the accuracy was not good enough to be used in the 3D estimator. See Figure 20 for an example of a human subject size.

**Figure 21:**
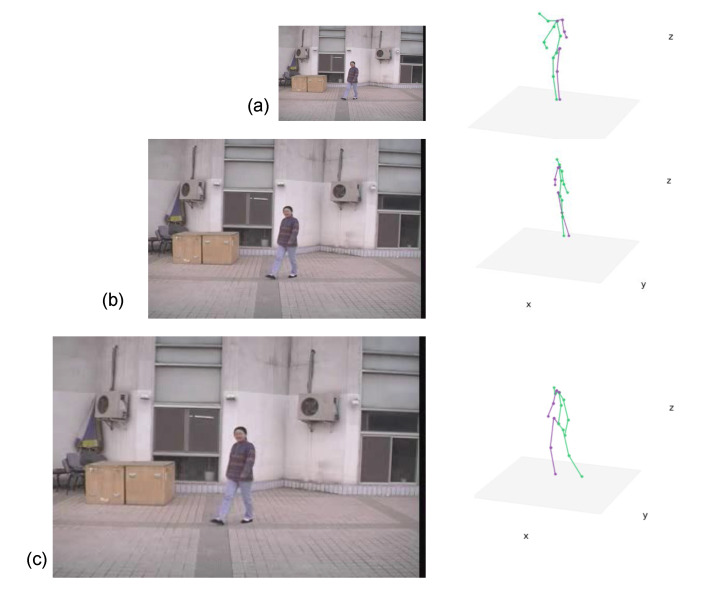
The test with scale-up images that shows a better 3D pose estimation. (**a**) The original image size 352×240. The 3D estimation result shows some deformation (**b**) The scaled image to 704×480. No more severe deformation is found. Yet, it is not enough to extract gait features (**c**) The scaled image to 1126×768. The human subject size is roughly 100×250 which is big enough as an input to 2D and 3D pose estimator.

We also conducted an additional qualitative experiment with a toy dataset to validate the low-resolution issue. See Figure 21 for more details. This test validates that the small human subject size in input images can be an important factor in estimation accuracy. Our future work will consider human subject size in the new training dataset.

Thus, as a future study, we plan to collect large-scale and high-definition gait-specific datasets and re-train 2D and 3D pose estimators with the newly acquired datasets to more fairly evaluate the gait feature-based approaches.

The potential limitations of the current work are as follows. According to [52], changing walking speed can be a significant factor in changing dorsiflexion of the knee and ankle. The authors in [52] found that peak values of the knee and ankle dorsiflexion in the side and front view of a subject significantly increased in fast walking compared with normal and slow walking. The proposed method assumes that the gait features of a subject share the same latent properties even at a different walking speed. The current study must be extended by conducting additional experiments to investigate the effect of different walking speeds in a specific subject’s gait features.

## 6. Conclusions

In this study, we proposed a markerless vision-based gait analysis technique and presented its validity by showing person re-identification performance using an individual’s gait patterns. We also conducted a comparative study of feature-based and spatiotemporal-based approaches to identify strengths and weaknesses. Our results indicate that using a recurrent neural network trained with spatiotemporal key joint values outperforms a feature-based approach. This study also suggests that gait feature-based methods need more accurate 2D and 3D key joint estimations. Our suggestion for future study is to develop large-scale human gait ground truth data to re-train 2D and 3D joint estimators to improve the inference quality of the estimators. Then we can expect that the proposed framework and comparative study will further contribute to rehabilitation studies, forensic gait analysis, and the early detection of neurological disorders.

## Figures and Tables

**Table 1:** The performance of classifiers for MARS and CASIA-A datasets.

	Random Forest		XGBoost		CATBoost		Siamese
	Acc.	F1	Acc.	F1	Acc.	F1	Acc.
MARS	81%	80%	82%	79%	83%	80%	99%
CASIA-A	32%	46%	41%	40%	34%	30%	95%

**Table 2:** The top 10 important features identified by the ensemble approaches.

	Random Forest	XGBoost	CATBoost
1	period	left_stride	period
2	right_stride	right_stride	right_stride
3	left_stride	period	left_upper_leg
4	Lfoof_period	left_upper_leg	left_stride
5	Rfoot_period	terminal_swing_ hip_extension	terminal_stance_ hip_extension
6	right_upper_leg	terminal_stance_ hip_extension	max_Ldegree
7	min_Ldegree	left_lower_leg	Lfoot_period
8	terminal_swing_ hip_extension	max_Ldegree	main_foot
9	min_Ldegree	left_lower_leg	Lfoot_period
10	terminal_stance_ hip_extension	right_upper_leg	terminal_swing_ hip_extension

## Data Availability

Not applicable.

## References

[1] Nixon M.S., Bouchrika I., Arbab-Zavar B., Carter J.N. On use of biometrics in forensics: Gait and ear. Proceedings of the 2010 18th European Signal Processing Conference.

[2] Liu Z., Zhang Z., Wu Q., Wang Y. (2015). Enhancing person re-identification by integrating gait biometric. Neurocomputing.

[3] Cuntoor K.R., Kale A., Rajagopalan A.N., Cuntoor N., Krüger V. Gait-based Recognition of Humans Using Continuous HMMs. Proceedings of the Fifth IEEE International Conference on Automatic Face and Gesture Recognition.

[4] Wang L., Tan T., Ning H., Hu W. (2003). Silhouette Analysis-Based Gait Recognition for Human Identification. IEEE Trans. Pattern Anal. Mach. Intell..

[5] Larsen P.K., Simonsen E.B., Lynnerup N. (2008). Gait Analysis in Forensic Medicine. J. Forensic Sci..

[6] Nixon M.S., Carter J.N. (2006). Automatic Recognition by Gait. Proc. IEEE.

[7] Han J., Bhanu B. (2006). Individual recognition using gait energy image. IEEE Trans. Pattern Anal. Mach. Intell..

[8] Colyer S.L., Evans M., Cosker D.P., Salo A.I.T. (2018). A Review of the Evolution of Vision-Based Motion Analysis and the Integration of Advanced Computer Vision Methods Towards Developing a Markerless System. Sport. Med.—Open.

[9] Latorre J., Colomer C., Alcañiz M., Llorens R. (2019). Gait analysis with the Kinect v2: Normative study with healthy individuals and comprehensive study of its sensitivity, validity, and reliability in individuals with stroke. J. Neuroeng. Rehabil..

[10] Andersson V.O., Araújo R.M. Person Identification Using Anthropometric and Gait Data from Kinect Sensor. Proceedings of the Twenty-Ninth AAAI Conference on Artificial Intelligence.

[11] Sinha A., Chakravarty K., Bhowmick B. Person Identification using Skeleton Information from Kinect. Proceedings of the Sixth International Conference on Advances in Computer-Human Interactions.

[12] Ahmed F., Paul P.P., Gavrilova M.L. (2015). DTW-based kernel and rank-level fusion for 3D gait recognition using Kinect. Vis. Comput..

[13] Jiang S., Wang Y., Zhang Y., Sun J., Jawahar C., Shan S. (2015). Real Time Gait Recognition System Based on Kinect Skeleton Feature. Computer Vision—ACCV 2014 Workshops.

[14] Sun J., Wang Y., Li J., Wan W., Cheng D., Zhang H. (2018). View-invariant gait recognition based on kinect skeleton feature. Multimed. Tools Appl..

[15] Yang F. (2018). Kinematics Research Progress of Swim-start on the New Start Block. Phys. Act. Health.

[16] Yao L., Kusakunniran W., Wu Q., Zhang J., Tang Z., Yang W. (2021). Robust gait recognition using hybrid descriptors based on Skeleton Gait Energy Image. Pattern Recognit. Lett..

[17] Das Choudhury S., Tjahjadi T. (2015). Robust view-invariant multiscale gait recognition. Pattern Recognit..

[18] Zeng W., Wang C. View-invariant gait recognition via deterministic learning. Proceedings of the 2014 International Joint Conference on Neural Networks (IJCNN).

[19] Bouchrika I., Nixon M.S., Gagalowicz A., Philips W. (2007). Model-Based Feature Extraction for Gait Analysis and Recognition. Computer Vision/Computer Graphics Collaboration Techniques.

[20] Krzeszowski T., Switonski A., Kwolek B., Josinski H., Wojciechowski K., Chmielewski L.J., Kozera R., Shin B.S., Wojciechowski K. (2014). DTW-Based Gait Recognition from Recovered 3-D Joint Angles and Inter-ankle Distance. Computer Vision and Graphics.

[21] Zheng L., Yang Y., Hauptmann A.G. (2016). Person Re-identification: Past, Present and Future. arXiv.

[22] Gray D., Brennan S., Tao H. Evaluating appearance models for recognition, reacquisition, and tracking. Proceedings of the IEEE International Workshop on Performance Evaluation for Tracking and Surveillance.

[23] Andriluka M., Pishchulin L., Gehler P.V., Schiele B. 2D Human Pose Estimation: New Benchmark and State of the Art Analysis. Proceedings of the 2014 IEEE Conference on Computer Vision and Pattern Recognition.

[24] Cao Z., Hidalgo Martinez G., Simon T., Wei S.E., Sheikh Y.A. (2021). OpenPose: Realtime Multi-Person 2D Pose Estimation using Part Affinity Fields. IEEE Trans. Pattern Anal. Mach. Intell..

[25] Newell A., Yang K., Deng J. Stacked Hourglass Networks for Human Pose Estimation. Proceedings of the 14th European Conference on Computer Vision.

[26] Pishchulin L., Insafutdinov E., Tang S., Andres B., Andriluka M., Gehler P., Schiele B. DeepCut: Joint Subset Partition and Labeling for Multi Person Pose Estimation. Proceedings of the 2016 IEEE Conference on Computer Vision and Pattern Recognition (CVPR).

[27] Insafutdinov E., Pishchulin L., Andres B., Andriluka M., Schiele B., Leibe B., Matas J., Sebe N., Welling M. (2016). DeeperCut: A Deeper, Stronger, and Faster Multi-person Pose Estimation Model. Computer Vision—ECCV 2016.

[28] Papandreou G., Zhu T., Kanazawa N., Toshev A., Tompson J., Bregler C., Murphy K. Towards Accurate Multi-person Pose Estimation in the Wild. Proceedings of the 2017 IEEE Conference on Computer Vision and Pattern Recognition (CVPR).

[29] Mehta D., Sotnychenko O., Mueller F., Xu W., Sridhar S., Pons-Moll G., Theobalt C. (2017). Single-Shot Multi-Person 3D Body Pose Estimation From Monocular RGB Input. arXiv.

[30] Taylor C. (2000). Reconstruction of Articulated Objects from Point Correspondences in a Single Uncalibrated Image. Comput. Vis. Image Underst..

[31] Iqbal U., Doering A., Yasin H., Krüger B., Weber A., Gall J. (2018). A dual-source approach for 3D human pose estimation from single images. Comput. Vis. Image Underst..

[32] Martinez J., Hossain R., Romero J., Little J.J. A Simple Yet Effective Baseline for 3d Human Pose Estimation. Proceedings of the 2017 IEEE International Conference on Computer Vision (ICCV).

[33] Wan Q., Zhang W., Xue X. (2017). DeepSkeleton: Skeleton Map for 3D Human Pose Regression. arXiv.

[34] Zhou X., Leonardos S., Hu X., Daniilidis K. 3D shape estimation from 2D landmarks: A convex relaxation approach. Proceedings of the 2015 IEEE Conference on Computer Vision and Pattern Recognition (CVPR).

[35] Rogez G., Weinzaepfel P., Schmid C. (2019). LCR-Net++: Multi-person 2D and 3D Pose Detection in Natural Images. IEEE Trans. Pattern Anal. Mach. Intell..

[36] Tome D., Russell C., Agapito L. Lifting from the Deep: Convolutional 3D Pose Estimation from a Single Image. Proceedings of the 2017 IEEE Conference on Computer Vision and Pattern Recognition (CVPR).

[37] Kudo Y., Ogaki K., Matsui Y., Odagiri Y. (2018). Unsupervised Adversarial Learning of 3D Human Pose from 2D Joint Locations. arXiv.

[38] Chen C., Ramanan D. 3D Human Pose Estimation = 2D Pose Estimation + Matching. Proceedings of the 2017 IEEE Conference on Computer Vision and Pattern Recognition (CVPR).

[39] Pezoa F., Reutter J.L., Suarez F., Ugarte M., Vrgoč D. Foundations of JSON schema. Proceedings of the 25th International Conference on World Wide Web.

[40] Ionescu C., Papava D., Olaru V., Sminchisescu C. (2014). Human3.6M: Large Scale Datasets and Predictive Methods for 3D Human Sensing in Natural Environments. IEEE Trans. Pattern Anal. Mach. Intell..

[41] Balazia M., Sojka P. (2018). Gait Recognition from Motion Capture Data. ACM Trans. Multimed. Comput. Commun. Appl..

[42] Ball A., Rye D., Ramos F., Velonaki M. (2012). Unsupervised clustering of people from ‘skeleton’ data. Proceedings of the Seventh Annual ACM/IEEE International Conference on Human-Robot Interaction.

[43] Breiman L. (2001). Random Forests. Mach. Learn..

[44] Chen T., Guestrin C. (2016). XGBoost: A Scalable Tree Boosting System. Proceedings of the 22nd ACM SIGKDD International Conference on Knowledge Discovery and Data Mining.

[45] Prokhorenkova L., Gusev G., Vorobev A., Dorogush A.V., Gulin A. (2018). CatBoost: Unbiased boosting with categorical features. Proceedings of the 32nd International Conference on Neural Information Processing Systems.

[46] Koch G., Zemel R., Salakhutdinov R. Siamese neural networks for one-shot image recognition. Proceedings of the ICML Deep Learning Workshop.

[47] Mueller J., Thyagarajan A. (2016). Siamese recurrent architectures for learning sentence similarity. Proceedings of the Thirtieth AAAI Conference on Artificial Intelligence.

[48] Zheng L., Bie Z., Sun Y., Wang J., Su C., Wang S., Tian Q. MARS: A Video Benchmark for Large-Scale Person Re-Identification. Proceedings of the 14th European Conference on Computer Vision—ECCV.

[49] Pedregosa F., Varoquaux G., Gramfort A., Michel V., Thirion B., Grisel O., Blondel M., Prettenhofer P., Weiss R., Dubourg V. (2011). Scikit-learn: Machine Learning in Python. J. Mach. Learn. Res..

[50] Kingma D.P., Ba J. Adam: A Method for Stochastic Optimization. Proceedings of the 3rd International Conference on Learning Representations, ICLR 2015.

[51] Van der Maaten L., Hinton G. (2008). Visualizing Data using t-SNE. J. Mach. Learn. Res..

[52] Sun D., Fekete G., Mei Q., Gu Y. (2018). The effect of walking speed on the foot inter-segment kinematics, ground reaction forces and lower limb joint moments. PeerJ.

